# Performance and hypothetical clinical impact of an mNGS-based machine learning model for antimicrobial susceptibility prediction of five ESKAPEE bacteria

**DOI:** 10.1128/spectrum.02592-24

**Published:** 2025-04-17

**Authors:** Yaoguang Li, Sizhen Liu, Peng Han, Jun Lei, Huifen Wang, Weiwei Zhu, Zihui Dong, Yize Zhang, Zhi Jiang, Beiwen Zheng, Guanhua Rao, Zujiang Yu, Ang Li

**Affiliations:** 1 Gene Hospital of Henan Province, Precision Medicine Center, the First Affiliated Hospital of Zhengzhou University, Zhengzhou, China; 2 Department of Infectious Disease, the First Affiliated Hospital of Zhengzhou University, Zhengzhou, China; 3 Genskey Medical Technology Co., Ltd., Beijing, China; 4 Collaborative Innovation Center for Diagnosis and Treatment of Infectious Diseases, State Key Laboratory for Diagnosis and Treatment of Infectious Diseases, The First Affiliated Hospital, Zhejiang University School of Medicine, Hangzhou, China; Children's National Hospital, George Washington University, Washington, DC, USA

**Keywords:** metagenomic next-generation sequencing, antimicrobial susceptibility testing, machine learning, genotypic antimicrobial susceptibility testing, ESKAPEE

## Abstract

Antimicrobial resistance is an escalating global health crisis, underscoring the urgent need for timely and targeted therapies to ensure effective clinical treatment. We developed a machine learning model based on metagenomic next-generation sequencing (mNGS) for rapid antimicrobial susceptibility prediction (mNGS-based AST), which was tailored to five ESKAPEE bacteria: *Acinetobacter baumannii*, *Klebsiella pneumoniae*, *Escherichia coli*, *Pseudomonas aeruginosa,* and *Staphylococcus aureus*. However, the clinical utility remained unvalidated. Assuming that mNGS-based AST results were obtained during clinical management, we assessed its clinical utility using data from a previous observational cohort study of clinical mNGS applications. We collected the data from 114 patients infected with five ESKAPEE bacteria from 07/2021 to 03/2023 and incorporated the sequencing data into the model. We evaluated the performance and hypothetical impact of the method by comparing its results and therapy recommendations with those based on traditional culture-based AST. The primary outcome was the performance of mNGS-based AST (*n* = 113 strains). mNGS-based AST displayed an overall accuracy of 93.84% and shorter turnaround time (1.12 ± 0.33 days vs 2.81 ± 0.57 days for culture-based AST, *t* = −27.31, *P* < 0.05). The secondary outcomes included the proportion of patients who could benefit from mNGS-based AST. It could allow earlier and suitable antibacterial adjustments in 32.05% of culture-positive patients (25/78) and offer actionable antimicrobial susceptibility results in 16.67% of culture-negative cases (6/36). mNGS-based AST offers a promising approach for individualized antibacterial therapy.

**IMPORTANCE:**

Metagenomic next-generation sequencing (mNGS)-based antimicrobial susceptibility prediction (AST) is a novel method for predicting the antimicrobial susceptibility of ESKAPEE bacteria using a machine learning approach and short-read sequencing data. Assuming that mNGS-based AST results were obtained during clinical management, it could significantly reduce turnaround time while maintaining a high level of accuracy, allowing for earlier therapeutic adjustments for patients. Furthermore, mNGS-based AST can be integrated with clinical mNGS to maximize the utility of short-read data without substantial cost increases. This study demonstrates the potential of mNGS-based AST for precise, individualized antibacterial selection and highlights its broader applicability in enhancing clinical antimicrobial use for various infections.

## INTRODUCTION

The emergence and spread of multidrug-resistant (MDR) bacteria pose a great threat to public health (1). A group of bacteria named “ESKAPE” by the Infectious Diseases Society of America, comprising *Enterococcus faecium* (“E”), *Staphylococcus aureus* (“S”, SA), *Klebsiella pneumoniae* (“K”, KP), *Acinetobacter baumannii* (“A”, AB), *Pseudomonas aeruginosa* (“P”, PA), and *Enterobacter* spp. (“E”), is responsible for most of the morbidity and mortality associated with antimicrobial-resistant bacteria (2). This concept was later expanded to “ESKAPEE” with the inclusion of *Escherichia coli* (EC) (3). Infections caused by MDR bacteria are associated with increased mortality and healthcare costs (4). Empirical treatment in the absence of etiological evidence often results in suboptimal therapeutic outcomes. Therefore, rapid identification of bacterial species and antimicrobial resistance is essential for effective clinical management (5).

Rapid antimicrobial susceptibility testing (AST) has attracted considerable interest, driving the development of both phenotypic and genotypic methods (6). Rapid phenotypic AST methods measure signals produced by bacterial growth and multiplication (7), but they are often limited in their ability to provide comprehensive resistance profiles. Recently, genotypic methods have become increasingly viable as our understanding of genotype–phenotype correlations has improved. Some sequencing-based genotypic methods generate extensive sequencing data, enabling identification of multiple resistance mutations. Whole-genome sequencing (WGS), in particular, offers detailed insights into resistance mutations and strain typing for tracking transmission (8). However, it requires bacterial culture and isolation, which is challenging for hard-to-culture organisms. Targeted enrichment-based WGS can capture genomes directly from clinical samples using specifically designed baits, but it relies on prior clinical suspicion of specific pathogens, which has limited application when assessing mixed or unknown infections (9).

Clinical metagenomic next-generation sequencing (mNGS) is an isolation-free and hypothesis-free pathogen detection method (10). Despite challenges like high cost, limited accessibility, and potential identification of irrelevant organisms, it offers high-throughput, broad-spectrum, and high-sensitivity pathogen detection, particularly useful for diagnosing polymicrobial infections, unknown pathogens, or low-abundance pathogens undetectable by conventional methods (11). Clinical mNGS primarily involves two processes: next-generation sequencing (NGS) and bioinformatics analysis. NGS generates extensive sequencing data, which not only enables pathogen identification but also has the potential to predict antimicrobial susceptibility. However, this potential has been limited by the low abundance of antimicrobial resistance genes (ARGs) directly obtained from clinical samples and the challenges in assembling short reads generated by NGS. To address these limitations, we employed machine learning. Recently, we developed a rapid mNGS-based approach for predicting the antimicrobial susceptibility of AB (12) using a machine learning model (mNGS-based AST) and generalized the approach to four other ESKAPEE species, namely, KP (13), PA (14), EC (15), and SA. However, its utility has yet to be validated in clinical cohorts.

In this study, we aimed to validate the performance and hypothetical clinical impact of this model in a cohort of patients infected with the five aforementioned bacteria. The primary outcome was the performance of the mNGS-based AST, evaluated in terms of the accuracy, time efficiency, and predictable ratio (the proportion of predictable cases to all cases), with respect to culture-based traditional AST (culture-based AST) of the confirmed bacterial strains. The secondary outcome was the hypothetical clinical impact of mNGS-based AST, including the proportion of patients for whom mNGS-based AST could allow earlier therapeutic adjustments compared to real-world clinical practices.

## MATERIALS AND METHODS

### Study design and population

We retrospectively reviewed the data from patients who received clinical mNGS, enrolled from a previous observational study of clinical mNGS applications at the First Affiliated Hospital of Zhengzhou University. Between July 2021 and March 2023, clinical specimens from 1,342 patients were subjected to paired bacterial culture and clinical mNGS. Among these, we identified 288 patients with positive clinical mNGS results for AB, KP, EC, PA, or SA. In this study, we incorporated the NGS sequencing data of clinical mNGS into the mNGS-based AST model for further bioinformatics analysis. A total of 176 patients with available mNGS-based AST results for bacteria of interest were initially included.

**Fig 1:**
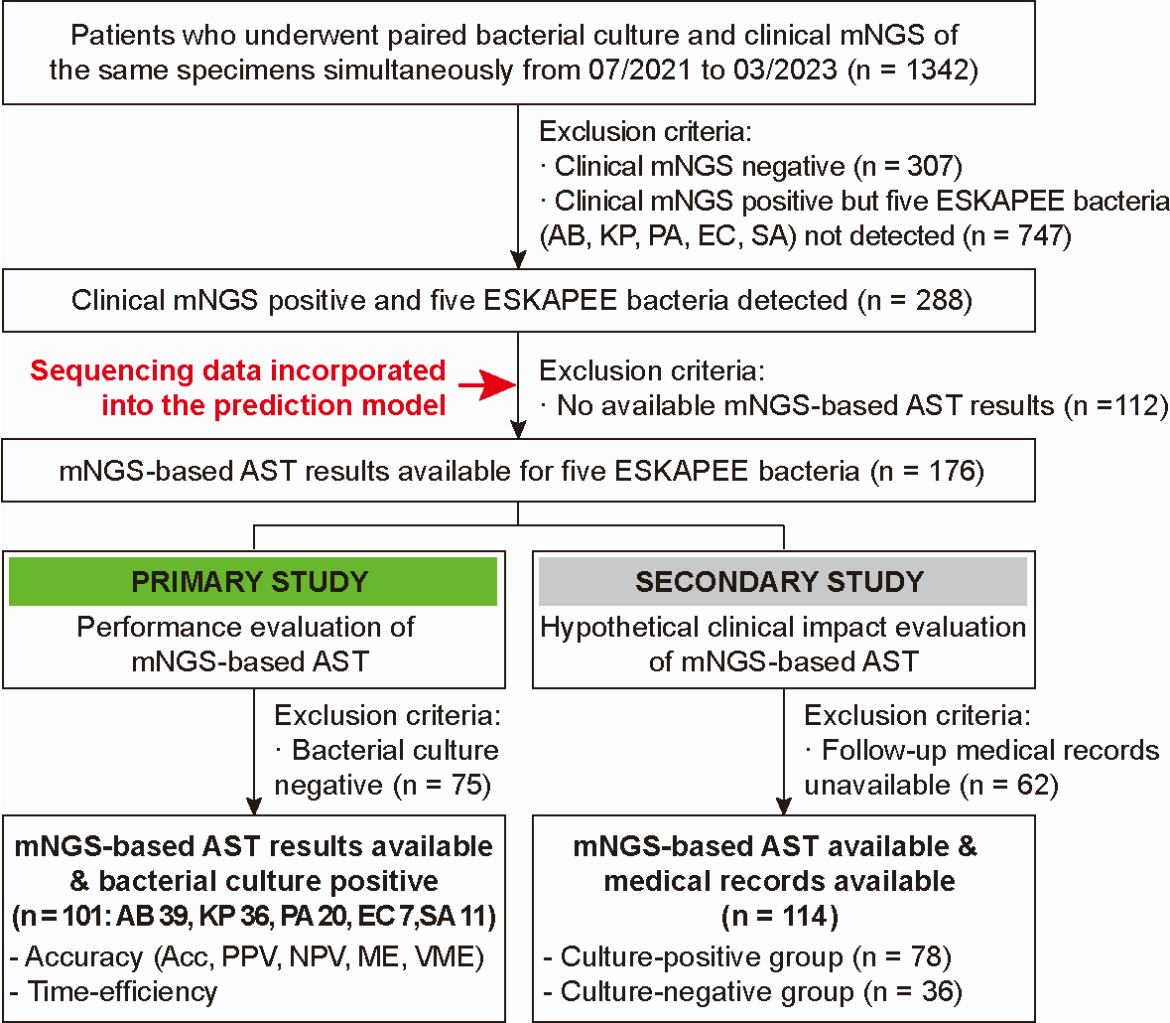
Study design and patient enrollment. The enrollment flow diagram depicts the evaluation of mNGS-based AST in patients infected with five ESKAPEE bacteria, from two perspectives: performance and hypothetical clinical impact. For patients with positive clinical mNGS results for the five ESKAPEE bacteria, sequencing data were integrated into the mNGS-based AST prediction model. Patients with negative bacterial culture results or incomplete medical records during follow-up were excluded. The patients were divided into two cohorts, which were subsequently used to assess the performance and hypothetical clinical impact of the mNGS-based AST model. Abbreviations: AB, *Acinetobacter baumannii*; Acc, accuracy; AST, antimicrobial susceptibility testing; EC, *Escherichia coli*; KP, *Klebsiella pneumoniae*; ME, major error; NPV, negative predictive value; PA, *Pseudomonas aeruginosa*; PPV, positive predictive value; SA, *Staphylococcus aureus*; VME, very major error.

Under the assumption that results of mNGS-based AST would typically be obtained during the clinical management of the patients, we evaluated the utility of the model for enrolled patients from two perspectives: performance and hypothetical clinical impact. For the primary study, antimicrobial susceptibility results of 113 confirmed bacterial strains (from 101 patients) were reviewed for performance evaluation. All of them had available results from both culture-based AST and mNGS-based AST. For the secondary study, we excluded patients whose medical records over the follow-up period (7 days) were incomplete. We yielded two cohorts according to bacterial culture results to evaluate the hypothetical clinical impact of the mNGS-based AST model, including the culture-positive group (*n* = 78) and the culture-negative group (*n* = 36). A schematic of the patient inclusion/exclusion workflow is shown in Fig. 1.

### Pathogen and antimicrobial susceptibility identification

#### Bacterial culture and culture-based AST

The Vitek 2 Compact system (bioMérieux, France) was used for bacterial identification and culture-based AST. Specifically, the antimicrobial susceptibility of the isolates was tested by the VITEK 2 system using AST-N335, AST-GN09, and AST-GP67 test cards (Oxoid, England) according to the Clinical and Laboratory Standards Institute (CLSI) (M100-S30 and M45-A3) and European Committee on Antimicrobial Susceptibility Testing (EUCAST) (v10.0) breakpoint tables, except for tigecycline and cefoperazone/sulbactam, for which the U.S. Food and Drug Administration (FDA) breakpoint tables were used. Antimicrobial susceptibility was reported as S (susceptible, susceptible at normal dosing), I (intermediate, susceptible at increased exposure), or R (resistant, resistant to the agent).

#### Clinical mNGS and mNGS-based AST

Clinical mNGS was conducted in two stages: sequencing and bioinformatics analysis. Sequencing was performed using the MGISEQ-200 platform (MGI, China) according to the manufacturer’s instructions. The resulting reads were processed through bioinformatics pipelines to identify potential pathogens. Human reads were removed by aligning to the GRCh38 reference assembly using Bowtie2 (v2.3.5.1). Remaining reads were mapped to a custom microorganism database with SNAP (v1.0) and taxonomically classified based on the NCBI (National Centre for Biotechnology Information) taxonomy annotations. Lastly, the identified microorganisms were cross-referenced with our clinical reportable range for a final filtering step to ensure clinical relevance.

**Fig 2:**
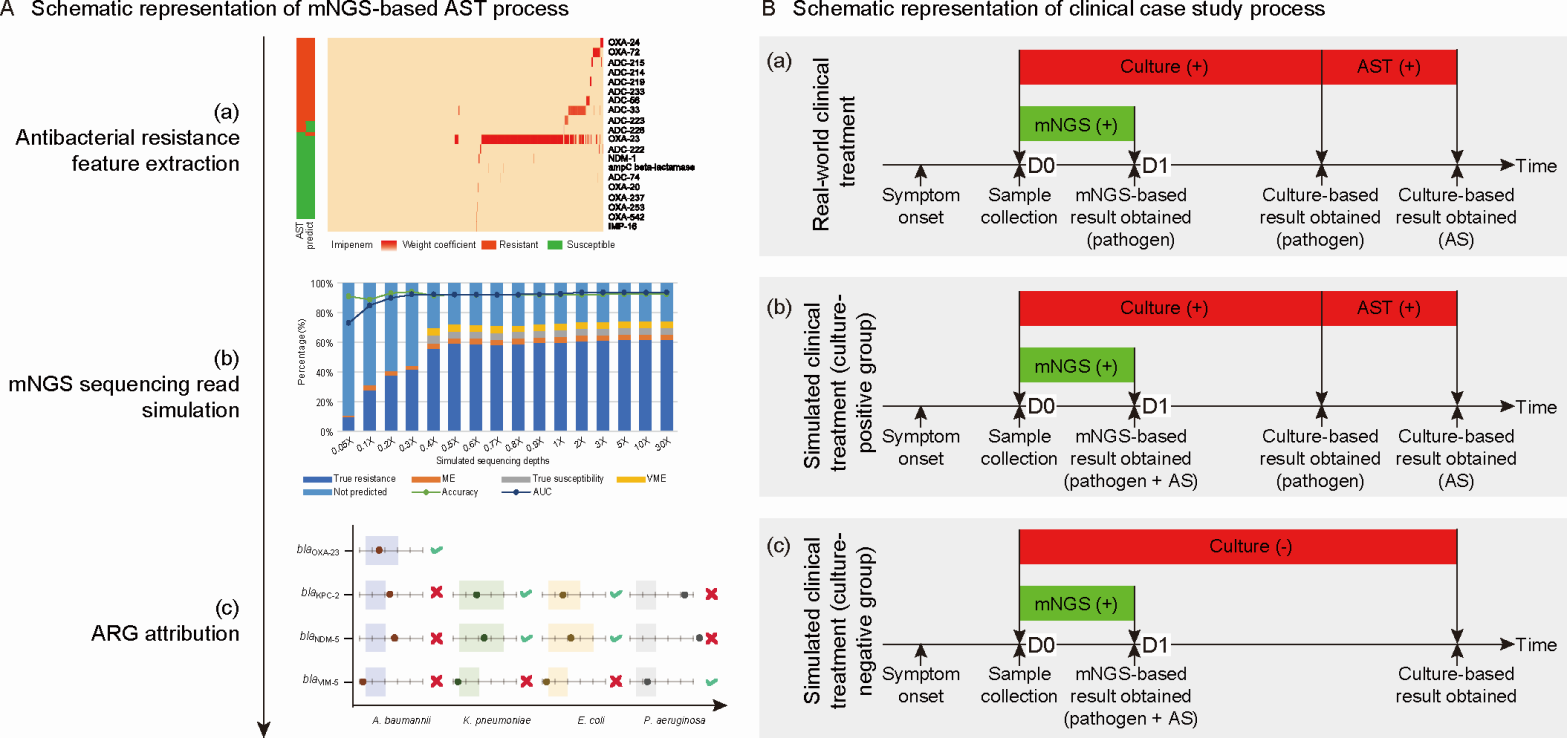
Schematic representations. (**A**) Schematic representation of the mNGS-based AST process, which consists of three main steps: (a) antibacterial resistance feature extraction; (b) mNGS sequencing read simulation; and (c) ARG attribution. (**B**) Schematic representation of the clinical case study process. (a) Real-world clinical treatment (culture-positive group). (b) Simulated clinical treatment (culture-positive group). Here, we assumed that mNGS-based AST results would be obtained along with clinical mNGS on D1. (c) Simulated clinical treatment (culture-negative group). This scenario is based on the same assumption as for the culture-positive group. Abbreviations: ARG, antimicrobial resistance gene; AS, antimicrobial susceptibility; AST, antimicrobial susceptibility testing.

mNGS-based AST leveraged the same sequencing stage as clinical mNGS. Then, the sequencing data were incorporated into the model to predict the antimicrobial susceptibility. Distinct models were formulated for each antibacterial–bacterium pair, and each model was constructed using the machine learning method, as reported previously (12–15). The initial prediction model was trained using culture-based AST results and WGS-based data. Least absolute shrinkage and selection operator (LASSO) regression was used to discover key genetic features significantly associated with antimicrobial resistance. After rigorous manual curation, the most optimal model—defined by the highest area under the curve (AUC) value—was selected for application. To apply the model for short-read sequencing data from mNGS, we simulated different read depths for pathogens to determine the necessary sequencing depth to fully cover the genomes and assess the accuracy of antimicrobial susceptibility predictions. Lastly, by employing strategies of species-specific k-mers in conjunction with ancillary methodologies, the key genetic features can be attributed to their corresponding pathogens (Fig. 2A; supplemental material).

In this study, we incorporated the sequencing data from prior clinical mNGS testing into the newly established prediction model. Pathogens that met the required sequencing depth threshold were included. If any resistance feature included in the optimal model was detected, the antimicrobial susceptibility was reported as “R-predicted.” If no such features were found, susceptibility determinations were based on the AUC value of the optimal model, using 0.9 as the threshold: for antibacterial–bacterium pairs with an AUC value exceeding 0.9, “S-predicted” was reported; otherwise, “Not-predicted” was reported.

### Clinical data collection

Clinical data of the enrolled patients were retrospectively collected through a review of the Electronic Medical Record System and Electronic Medical Prescription System at the First Affiliated Hospital of Zhengzhou University. The following information was obtained: (i) general hospitalization details, including age, sex, underlying diseases, infection site, and intensive care unit history; (ii) routine laboratory results, including the levels of white blood cell count (WBC), neutrophil count (NEUT), C-reactive protein (CRP), and procalcitonin (PCT); (iii) microbiological examination results and turnaround times, including bacterial culture, culture-based AST, and clinical mNGS results; and (iv) treatment data, specifically antibacterial use on the day of sampling (D0) and during the following 7 days (D1–D7).

### Performance and hypothetical clinical impact evaluation

#### Primary study: performance evaluation

The results of culture-based AST, initially reported as a trichotomous outcome (S, I, or R, as indicated above), were converted to dichotomous results (S or I/R) (16) to facilitate comparisons with the dichotomous outcomes from mNGS-based AST (S-predicted or R-predicted). When both methods indicated susceptibility to a particular antimicrobial agent for a specific strain (S vs S-predicted), this agreement was labeled as true susceptibility. Similarly, when both methods indicated a lack of susceptibility (I/R vs R-predicted), this concordance was labeled as true resistance. In cases of discrepancy, major error (ME, S vs R-predicted) and very major error (VME, I/R vs S-predicted) were defined to indicate the cases. If mNGS-based AST failed to generate the antimicrobial susceptibility results to an antimicrobial, the item was classified as Unpredicted. Entries were further categorized as Unpredicted-S or Unpredicted-R/I based on the culture-based AST results.

Using culture-based AST results as the gold standard, we evaluated the performance of mNGS-based AST. For the items whose results were available from mNGS-based AST, we calculated the accuracy, positive predictive value (PPV), and negative predictive value (NPV). The accuracy was defined as the proportion of true resistance and true susceptibility items to predictable items. PPV was defined as the proportion of true resistance items to the sum of true resistance and ME items. NPV was defined as the proportion of true susceptibility items to the sum of true susceptibility and VME items. For all items, we calculated the predictable ratio, defined as the proportion of predictable cases to the total number of items.

Additionally, the turnaround time of clinical mNGS (between sampling and obtaining available reports) was collected from the Electronic Medical Record System of the First Affiliated Hospital of Zhengzhou University. The turnaround time for mNGS-based AST was considered equivalent to that of clinical mNGS. The differences in the turnaround time between mNGS-based and culture-based AST were assessed.

#### Secondary study: hypothetical clinical impact evaluation

During real-world clinical management, specimens were collected and sent for bacterial culture and clinical mNGS on D0. Upon receiving clinical mNGS results on D1, clinicians could make initial adjustments referring to the mNGS findings. Further adjustments were made from D2 to D7, incorporating bacterial culture results and patient response over this period (Fig. 2Ba).

We aimed to assess the clinical benefits of the model in guiding therapy adjustments. To achieve this, we conducted a simulated management experiment, assuming that mNGS-based AST results were available on D1 along with clinical mNGS results. We analyzed the model’s hypothetical clinical impact based on recommendations for the antibacterial therapy administered to patients on D1. Two experienced physicians provided therapeutic recommendations independently based on mNGS-based AST or culture-based AST. These recommendations were categorized into three types: de-escalation (switching to narrower-spectrum antibacterials), escalation (switching to broader-spectrum antibacterials), and maintenance (continuing the current therapy). The cases with inconsistent judgments were re-evaluated through a case review process.

In simulated management, we primarily assessed whether the confirmed pathogenic bacterial strains were resistant to antibacterials administered on D1 according to mNGS-based AST or culture-based AST results. For patients in the culture-positive group, we compared the physicians’ recommendations based on each method. Cases where the recommendations were consistent were considered to demonstrate that mNGS-based AST had a positive clinical impact as mNGS-based AST enabled earlier therapy adjustments (D1 vs D2-7) (Fig. 2Bb). For patients in the culture-negative group, we assessed the proportion of those who could receive available recommendations despite the absence of culture-based results (Fig. 2Bc).

#### Statistical analysis

Continuous variables are expressed as median (interquartile range, IQR) or mean (standard deviation, SD). For normally distributed variables, independent *t*-test was performed with a significance level set at α = 0.05. Categorical variables are presented as frequencies and percentages. Statistical analysis was conducted using SPSS (version 28.0, IBM Corp. USA).

## RESULTS

### Study strains and cohort

Of the 1,342 patients with paired bacterial culture and clinical mNGS results, the five ESKAPEE bacteria were detected in clinical specimens of 288 patients by clinical mNGS, and mNGS-based AST results could be obtained for 176 of them (Fig. 1).

To assess the performance of mNGS-based AST, we included 113 culture-confirmed bacterial strains from 101 patients, comprising 39 strains of AB, 36 strains of KP, 20 strains of PA, 7 strains of EC, and 11 strains of SA.

To evaluate the hypothetical clinical impact of mNGS-based AST, we included 114 patients with available medical records during the 7-day follow-up period, comprising 78 patients in the culture-positive group and 36 in the culture-negative group. Of these patients, 79 (69.30%) were male, and the median age was 54 years, with a range of 8 to 86 years. Almost all patients (101, 94.74%) had underlying diseases, the most common being central nervous system diseases (42.98%), followed by cardiovascular diseases (28.95%). Sixteen patients (14.04%) had experienced trauma within 1 month prior to D0, and 27 (23.68%) had undergone surgery. Most patients (99, 86.84%) had received antibacterial agents prior to D0, and 78 patients (68.42%) were treated in the intensive care unit. Considering both the etiological test results and clinical symptoms, 73 patients (64.04%) were diagnosed with lower respiratory tract infections, 18 patients (15.79%) with central nervous system infections, and 11 patients (9.65%) with bloodstream infections (Table 1).

**TABLE 1:** Patient demographics and treatment overview

Demographic	N (%)/M (P25, P75)		
	Culture-positive group (*n* = 78)	Culture-negative group (*n* = 36)	Total (*n* = 114)
Male	55 (70.51)	24 (66.67)	79 (69.30)
Age (year)	55 (38.5, 63.0)	53.0 (34.8, 63.8)	54.0 (38.5, 63.0)
Underlying diseases			
Central nervous system diseases	33 (42.31)	16 (44.44)	49 (42.98)
Cardiovascular diseases	19 (24.36)	14 (38.89)	33 (28.95)
Respiratory diseases	12 (15.38)	6 (16.67)	18 (15.79)
Metabolic diseases	12 (15.38)	6 (16.67)	18 (15.79)
Digestive system diseases	8 (10.26)	4 (11.11)	12 (10.53)
Malignant hematological diseases	5 (6.41)	3 (8.33)	8 (7.02)
Kidney and urinary diseases	4 (5.13)	3 (8.33)	7 (6.14)
Trauma (within 1 month before D0)	14 (17.95)	2 (5.56)	16 (14.04)
Surgical history (within 1 month before D0)	25 (32.05)	2 (5.56)	27 (23.68)
Infection site			
Lower respiratory tract infection	57 (73.08)	16 (44.44)	73 (64.04)
Central nervous system infection	5 (6.41)	13 (36.11)	18 (15.79)
Bloodstream infection	5 (6.41)	6 (16.67)	11 (9.65)
Skin and soft tissue infection	9 (11.54)	0 (0.00)	9 (7.89)
Abdominal infection	1 (1.28)	1 (2.78)	2 (1.75)
Urinary tract infection	1 (1.28)	0 (0.00)	1 (0.88)
Routine test results (on D0)			
WBC (×10^9^ /L) (3.5–9.5)	9.60 (6.95, 14.78)	8.37 (5.07, 12.34)	9.49 (6.62, 14.24)
NEUT (×10^9^ /L) (1.8–6.3)	8.52 (5.18, 13.25)	5.74 (3.18, 11.18)	7.66 (4.28, 12.68)
CRP (mg/L) (0–5)	109.99 (30.48, 162.11)	67.20 (19.48, 158.19)	105.14 (24.68, 160.89)
PCT (ng/mL) (0–0.064)	0.527 (0.167, 2.275)	0.280 (0.092, 2.998)	0.52 (0.12, 2.31)
Antibacterial history before D0	67 (85.90)	32 (88.89)	99 (86.84)
Intensive care unit history before D0	53 (67.95)	25 (69.44)	78 (68.42)

### Primary outcomes: performance of mNGS-based AST

#### Accuracy

Compared with the results of culture-based AST, which served as the gold standard, the overall accuracy of mNGS-based AST was 93.84%. Furthermore, the overall PPV was 95.03%, and the overall NPV was 85.71% (Table S1). Additionally, mNGS-based AST could provide detailed data of drug resistance genes (Table S2).

**Fig 3:**
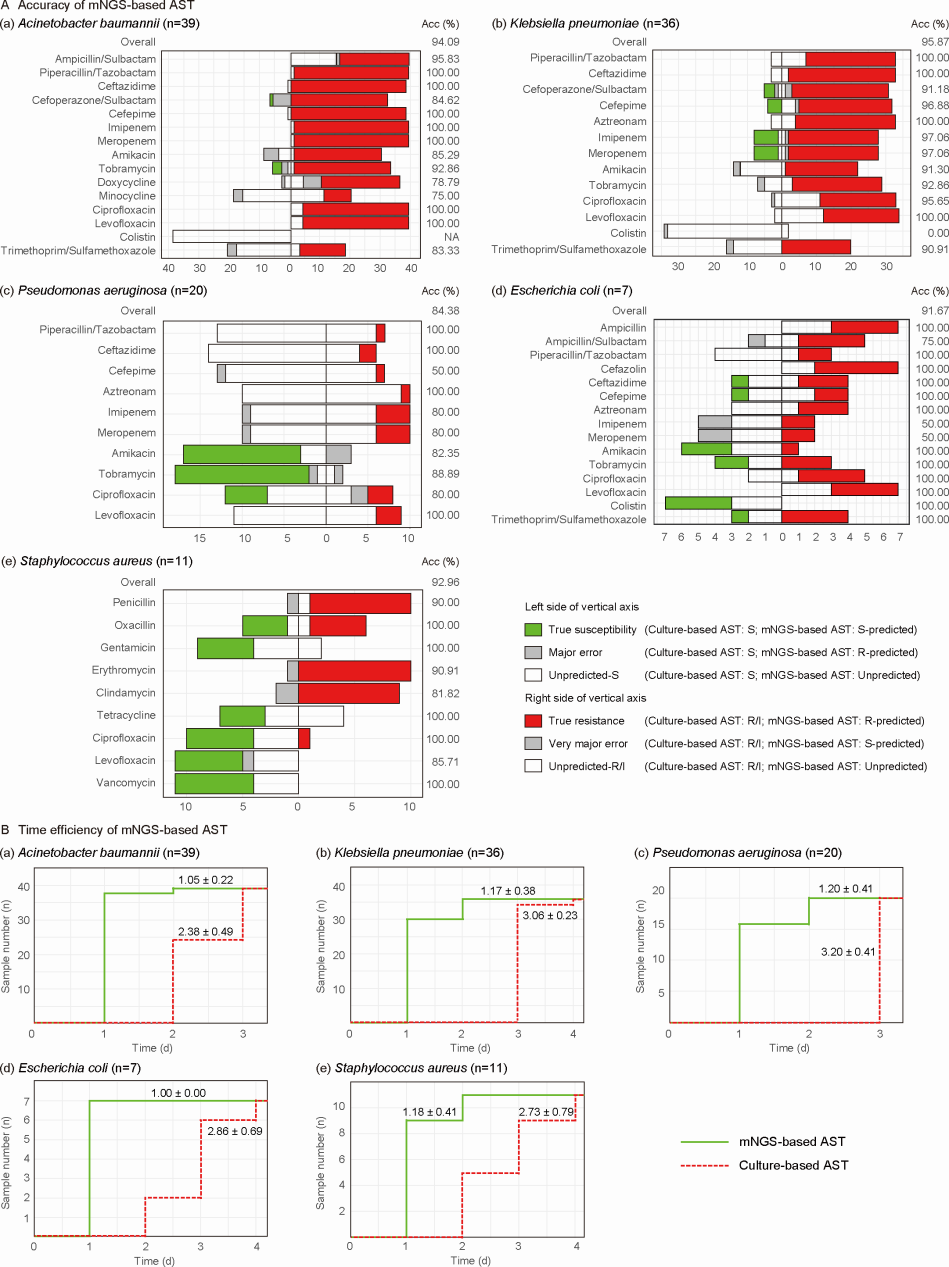
(A) Performance of mNGS-based AST accuracy of mNGS-based AST for five ESKAPE bacterial species and specific antimicrobial agents: (a) *Acinetobacter baumannii* (*n* = 39); (b) *Klebsiella pneumoniae* (*n* = 36); (c) *Pseudomonas aeruginosa* (*n* = 20); (d) *Escherichia coli* (*n* = 7); and (e) *Staphylococcus aureus* (*n* = 11). (**B**) Time efficiency of mNGS-based AST for five ESKAPE bacterial species and specific antimicrobial agents: (a) *Acinetobacter baumannii* (*n* = 39); (b) *Klebsiella pneumoniae* (*n* = 36); (c) *Pseudomonas aeruginosa* (*n* = 20); (d) *Escherichia coli* (*n* = 7); and (e) *Staphylococcus aureus* (*n* = 11). Abbreviations: Acc, accuracy; AST, antimicrobial susceptibility testing; I, intermediate; R, resistant; S, susceptible.

At the bacterium-specific level, the accuracy of mNGS-based AST was as follows: AB, 94.09%; KP, 95.87%; PA, 84.38%; EC, 91.67%; and SA, 92.96% (Table S3). Graphs of the antimicrobial susceptibility analysis for each bacterium are shown in Fig. 3A.

At the antibacterial-specific level, the accuracy of mNGS-based AST varied as follows, listed in the descending and alphabetical order: ampicillin, aztreonam, cefazolin, ceftazidime, gentamicin, oxacillin, piperacillin/tazobactam, tetracycline, and vancomycin, all at 100.00%; levofloxacin, 98.59%; cefepime, 97.33%; ciprofloxacin, 96.20%; imipenem, 95.06%; meropenem, 95.06%; tobramycin, 93.18%; ampicillin/sulbactam, 93.10%; erythromycin, 90.91%; penicillin, 90.00%; trimethoprim/sulfamethoxazole, 88.89%; cefoperazone/sulbactam, 87.67%; amikacin, 87.18%; clindamycin, 81.82%; colistin, 80.00%; doxycycline, 78.79%; minocycline, 75.00% (Table S4).

#### Time efficiency

Overall, mNGS-based AST had a significantly shorter turnaround time than culture-based AST (1.12 ± 0.33 days vs 2.81 ± 0.57 days, respectively; *t* = −27.31, *P* < 0.05).

Specifically, turnaround time for obtaining mNGS-based AST demonstrated superior efficiency at the individual bacterial level. The comparisons are as follows: AB (1.05 ± 0.22 days for mNGS-based AST vs 2.38 ± 0.49 days for culture-based AST; *t* = −15.39, *P* < 0.05), KP (1.17 ± 0.38 days vs 3.06 ± 0.23 days; *t* = −25.55, *P* < 0.05), PA (1.20 ± 0.41 days vs 3.20 ± 0.41 days; *t* = −15.41, *P* < 0.05), EC (1.00 ± 0.00 days vs 2.86 ± 0.69 days; *t* = −7.12, *P* < 0.05), and SA (1.18 ± 0.41 days vs 2.73 ± 0.79 days; *t* = −5.80, *P* < 0.05) (Fig. 3B; Table S5).

#### Predictable ratio

The overall predictable ratio of mNGS-based AST was 68.02%. Specifically, the ratio was 83.42% for items classified as R or I by culture-based AST and 33.56% for those classified as S (Table S1).

At the bacterium-specific level, the predictable ratio was as follows: AB, 78.12%; KP, 72.44%; PA, 32.00%; EC, 57.14%; and SA, 71.72% (Table S3). At the antibacterial-specific level, colistin had the lowest predictable ratio, at only 6.10% (Table S4).

### Secondary outcome: hypothetical clinical impact of mNGS-based AST

**Fig 4:**
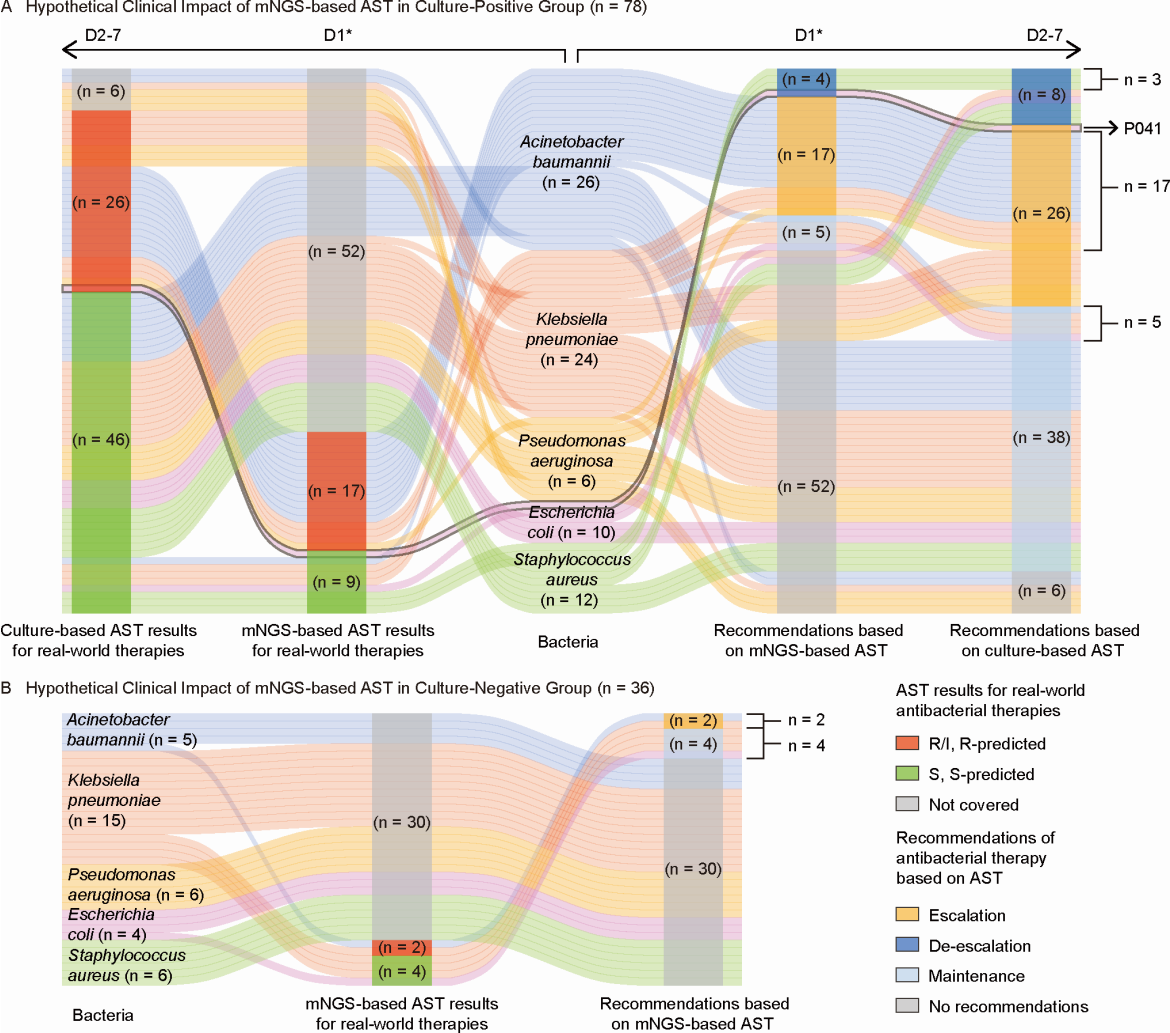
Hypothetical clinical impact of mNGS-based AST * On D1, in the simulated management scenario, mNGS-based AST results would be obtained along with clinical mNGS. (**A**) Hypothetical clinical impact of mNGS-based AST in the culture-positive group (*n* = 78). Each line, spanning from left to right, represents a single patient case, connecting the AST results and the corresponding therapy recommendations. To the left of the bacteria column, the columns represent whether the results of each method (culture-based AST and mNGS-based AST) could cover real-world antibacterial therapy on D1. To the right, the columns indicate antibacterial adjustment recommendations based on each method. (**B**) Hypothetical clinical impact of mNGS-based AST in the culture-negative group (*n* = 36). This diagram follows a similar structure to Part A. Differently, there are no culture-based results or corresponding recommendations. Abbreviations: I, intermediate; R, resistant; S, susceptible.

In the simulated management scenario of 78 patients in the culture-positive group, mNGS-based AST results could be obtained on D1 along with clinical mNGS, which could cover the real-world antibacterial use on D1 for 26 patients (33.33%): 17 cases were identified as using resistant antibacterials, while nine cases were found to be on susceptible antibacterials. According to mNGS-based AST, 17 patients (21.79%) could receive escalation advice, three patients (3.85%) could receive proper de-escalation advice, and five patients (6.41%) could receive advice to maintain the therapy. However, for one patient (P041), the carbapenem susceptibility predicted by mNGS-based AST was S-predicted, in contrast to the culture-based AST, which could have a negative impact on clinical management. mNGS-based AST could allow earlier therapy adjustments in 32.05% (25/78) culture-positive patients. The consistency between the recommendations based on mNGS-based AST and culture-based AST was 96.15% (25/26) (Fig. 4A).

In the simulated management scenario of 36 patients in the culture-negative group, mNGS-based results could cover real-world antibacterial use on D1 for 6 patients (16.67%): two cases were identified as using resistant antibacterials, while four cases were found to be on susceptible antibacterials. mNGS-based AST could provide available recommendations for antibacterial adjustment in six patients (16.67%): escalation for two patients (5.56%), and maintenance for four patients (11.11%) (Fig. 4B).

## DISCUSSION

Bacterial resistance, particularly among ESKAPEE bacteria, poses a critical global health challenge due to its severe impact on therapeutic effects for life-threatening infections (17). Therefore, rapid and accurate identification of antimicrobial susceptibility is increasingly vital for guiding appropriate therapy in cases of infections.

In this study, we evaluated the performance and hypothetical clinical impact of mNGS-based AST using data from a clinical cohort. To the best of our knowledge, this is the first study to assess the applicability of an mNGS-based machine-learning approach for AST in managing patients infected with ESKAPEE bacteria.

Compared to culture-based AST, mNGS-based AST performed satisfactorily in terms of both accuracy (93.84% overall) and turnaround time (average 1.12 ± 0.33 days). This indicates that the use of mNGS-based AST can significantly reduce turnaround time while maintaining a high level of accuracy. However, several concerns require attention. First, the predictable ratio was low, especially for PA (32.00%) and for colistin (6.10%). Without pathogen isolation, mNGS-generated sequences from clinical specimens contained a large number of human-derived sequences (18, 19), which hampers the detection of microbial-derived ARGs. Therefore, many antibacterial–bacterium pairs were classified as “Unpredicted.” Second, the performance varied across different bacteria and antibacterial agents; notably, the accuracy for PA was relatively limited (84.38%). The low accuracy and predictable ratio for PA align with the findings from previous AST studies, primarily due to the complex antimicrobial resistance mechanisms in this species. These mechanisms involve synergistic effects between multiple energy-dependent active drug efflux pumps and low-permeability outer membrane barriers, posing significant challenges for genotypic AST (20). Moreover, the model underperformed in predicting susceptibility to colistin, partly because gram-negative bacteria globally show low rates of resistance to it, and the mechanisms behind colistin remain poorly understood (21). Deeper insights into resistance mechanisms are essential for improving the model. Simultaneously, mNGS-based AST results are less accessible in the antibacterial–bacterium pairs classified as S by culture-based AST, as reflected in the overall NPV (85.71% vs 95.03% for PPV) and a lower predictable ratio (33.56% vs 83.42% in items classified as R/I). This lower performance in susceptible items is due to the limited number of “S-predicted” cases because of the cautious approach we took in reporting “S-predicted” results and inadequate microbial-derived data from clinical specimens. Additionally, the algorithm requires further refinement. In the case of P041, the process incorrectly attributed ARGs from EC to *Enterococcus faecium*, resulting in errors in the prediction results and indicating the need to improve the species assignment accuracy of the model, as well as its ARG identification capability.

We also obtained evidence that the mNGS-based AST model could positively influence the clinical management of patients infected with ESKAPEE bacterial species. On the one hand, mNGS-based AST facilitates earlier therapeutic adjustments in more cases, owing to its shorter turnaround compared to culture-based AST and its applicability in some culture-negative cases. In the culture-positive group, mNGS-based AST could provide actionable predictions for antibacterial adjustment in 26 patients, with 25 aligning with culture-based AST (except for patient P041). Notably, mNGS-based AST could enable earlier targeted therapy adjustments in 32.05% (25/78) of patients. For culture-negative patients, mNGS-based AST could provide available antimicrobial susceptibility results in 16.67% of cases. For patients with a high suspicion of infection but negative cultures, clinicians typically repeat the bacterial culture to increase the likelihood of positive identification (22); however, this approach is time-consuming and offers limited additional information. In this regard, clinical mNGS and mNGS-based AST can serve as second-line options in culture-negative cases (23). In the absence of culture-based AST confirmation, mNGS-based AST delivers valuable insights into antimicrobial susceptibility. On the other hand, mNGS-based AST complements clinical mNGS, providing more precise medication recommendations. In the real-world clinical treatment, clinicians received clinical mNGS results and made initial adjustments with reference to the results on D1. Therefore, the therapy recommendations based on mNGS-based AST to antibacterial use on D1 can be seen as a complement to clinical mNGS. By expanding and supplementing the information provided by clinical mNGS, mNGS-based AST greatly simplifies the report interpretation and enhances the ability of mNGS technology to guide decision-making in treatment.

Our study has several limitations. First, it is a single-center study with a small sample size (113 confirmed bacterial strains and 114 patients) from the First Affiliated Hospital of Zhengzhou University. The high rate of antibacterial history and intensive care unit history in this cohort serves as sources of potential bias, limiting the generalizability of our findings, particularly regarding the hypothetical clinical impact. Therefore, multicenter studies with larger sample sizes should be conducted. Second, we compared mNGS-based AST results with those from the VITEK system, which generated results by comparing timed turbidity measurements to a predetermined threshold. While widely used and reliable, the VITEK system does not always capture true antimicrobial susceptibility, and differences may introduce statistical uncertainty in accuracy assessments. Future studies should re-evaluate the mNGS-based AST model using methods like broth microdilution. Third, our models could not provide minimum inhibitory concentration (MIC) values. In clinical practice, when using antibacterials like β-lactams, aminoglycosides, and quinolones—the specific MIC value plays a pivotal role in determining the treatment regimen—currently, we recommend clinicians first choose susceptible antibacterials based on mNGS-based AST and adjust dosages after getting culture-based AST results. Looking ahead, a sufficient amount of reliable MIC data may be helpful for further optimizing the model to provide an MIC-like index. Lastly, the current mNGS-based AST model cannot make predictions for tigecycline, a commonly used antibacterial agent in our institution, which also necessitates further model improvement and integration. This also suggests that incorporating geographically specific patterns into the model would enable it to account for differences in treatment protocols across clinical facilities, improving the accuracy of individualized susceptibility predictions.

### Conclusions

In conclusion, mNGS-based AST shows satisfactory performance and hypothetical clinical impact in a clinical cohort infected by five ESKAPEE bacteria, offering a promising approach for individualized antibacterial therapy. Furthermore, this approach holds potential for broader application to additional microbial species, providing valuable guidance for precise antimicrobial use.

## Data Availability

The raw sequence data for this study have been uploaded and deposited in NCBI/ENA/DDBJ under BioProject no. PRJNA1134399. The data analyzed during this study are included in the supplemental material. More deidentified data are available from corresponding author Ang Li (lia@zju.edu.cn) upon reasonable request.

## SUPPLEMENTAL MATERIAL

Supplemental Material spectrum.02592-24-s0001.pdf Supplemental methods; legends for Tables S1 to S5.

Supplemental Tables spectrum.02592-24-s0002.xlsx Tables S1 to S5.

ASM does not own the copyrights to Supplemental Material that may be linked to, or accessed through, an article. The authors have granted ASM a non-exclusive, world-wide license to publish the Supplemental Material files. Please contact the corresponding author directly for reuse.

## References

[1] Catalano A, Iacopetta D, Ceramella J, Pellegrino M, Giuzio F, Marra M, Rosano C, Saturnino C, Sinicropi MS, Aquaro S. 2023. Antibiotic-resistant ESKAPE pathogens and COVID-19: the pandemic beyond the pandemic. Viruses 15:1843. doi:10.3390/v15091843 37766250 PMC10537211

[2] Miller WR, Arias CA. 2024. ESKAPE pathogens: antimicrobial resistance, epidemiology, clinical impact and therapeutics. Nat Rev Microbiol 22:598–616. doi:10.1038/s41579-024-01054-w 38831030 PMC13147291

[3] Partridge SR, Kwong SM, Firth N, Jensen SO. 2018. Mobile genetic elements associated with antimicrobial resistance. Clin Microbiol Rev 31:e00088-17. doi:10.1128/CMR.00088-17 30068738 PMC6148190

[4] Jernigan JA, Hatfield KM, Wolford H, Nelson RE, Olubajo B, Reddy SC, McCarthy N, Paul P, McDonald LC, Kallen A, Fiore A, Craig M, Baggs J. 2020. Multidrug-resistant bacterial infections in U.S. Hospitalized patients, 2012-2017. N Engl J Med 382:1309–1319. doi:10.1056/NEJMoa1914433 32242356 PMC10961699

[5] De Oliveira DMP, Forde BM, Kidd TJ, Harris PNA, Schembri MA, Beatson SA, Paterson DL, Walker MJ. 2020. Antimicrobial resistance in ESKAPE pathogens. Clin Microbiol Rev 33:e00181-19. doi:10.1128/CMR.00181-19 32404435 PMC7227449

[6] van Belkum A, Burnham C-A, Rossen JWA, Mallard F, Rochas O, Dunne WM Jr. 2020. Innovative and rapid antimicrobial susceptibility testing systems. Nat Rev Microbiol 18:299–311. doi:10.1038/s41579-020-0327-x 32055026

[7] Behera B, Anil Vishnu GK, Chatterjee S, Sitaramgupta V VSN, Sreekumar N, Nagabhushan A, Rajendran N, Prathik BH, Pandya HJ. 2019. Emerging technologies for antibiotic susceptibility testing. Biosens Bioelectron 142:111552. doi:10.1016/j.bios.2019.111552 31421358

[8] Casali N, Nikolayevskyy V, Balabanova Y, Harris SR, Ignatyeva O, Kontsevaya I, Corander J, Bryant J, Parkhill J, Nejentsev S, Horstmann RD, Brown T, Drobniewski F. 2014. Evolution and transmission of drug-resistant tuberculosis in a Russian population. Nat Genet 46:279–286. doi:10.1038/ng.2878 24464101 PMC3939361

[9] Brown AC, Bryant JM, Einer-Jensen K, Holdstock J, Houniet DT, Chan JZM, Depledge DP, Nikolayevskyy V, Broda A, Stone MJ, Christiansen MT, Williams R, McAndrew MB, Tutill H, Brown J, Melzer M, Rosmarin C, McHugh TD, Shorten RJ, Drobniewski F, Speight G, Breuer J. 2015. Rapid whole-genome sequencing of Mycobacterium tuberculosis isolates directly from clinical samples. J Clin Microbiol 53:2230–2237. doi:10.1128/JCM.00486-15 25972414 PMC4473240

[10] Piantadosi A, Mukerji SS, Ye S, Leone MJ, Freimark LM, Park D, Adams G, Lemieux J, Kanjilal S, Solomon IH, Ahmed AA, Goldstein R, Ganesh V, Ostrem B, Cummins KC, Thon JM, Kinsella CM, Rosenberg E, Frosch MP, Goldberg MB, Cho TA, Sabeti P. 2021. Enhanced virus detection and metagenomic sequencing in patients with meningitis and encephalitis. mBio 12:e0114321. doi:10.1128/mBio.01143-21 34465023 PMC8406231

[11] Liu BM, Mulkey SB, Campos JM, DeBiasi RL. 2024. Laboratory diagnosis of CNS infections in children due to emerging and re-emerging neurotropic viruses. Pediatr Res 95:543–550. doi:10.1038/s41390-023-02930-6 38042947 PMC12494120

[12] Hu X, Zhao Y, Han P, Liu S, Liu W, Mai C, Deng Q, Ren J, Luo J, Chen F, Jia X, Zhang J, Rao G, Gu B. 2023. Novel clinical mNGS-based machine learning model for rapid antimicrobial susceptibility testing of Acinetobacter baumannii. J Clin Microbiol 61:e0180522. doi:10.1128/jcm.01805-22 37022167 PMC10204632

[13] Xu Y, Liu D, Han P, Wang H, Wang S, Gao J, Chen F, Zhou X, Deng K, Luo J, Zhou M, Kuang D, Yang F, Jiang Z, Xu S-H, Rao G, Wang Y, Qu J. 2024. Rapid inference of antibiotic resistance and susceptibility for Klebsiella pneumoniae by clinical shotgun metagenomic sequencing. Int J Antimicrob Agents 64:107252. doi:10.1016/j.ijantimicag.2024.107252 38908534

[14] Liu B, Gao J, Liu XF, Rao G, Luo J, Han P, Hu W, Zhang Z, Zhao Q, Han L, Jiang Z, Zhou M. 2023. Direct prediction of carbapenem resistance in Pseudomonas aeruginosa by whole genome sequencing and metagenomic sequencing. J Clin Microbiol 61:e0061723. doi:10.1128/jcm.00617-23 37823665 PMC10662344

[15] Tian Y, Zhang D, Chen F, Rao G, Zhang Y. 2024. Machine learning-based colistin resistance marker screening and phenotype prediction in Escherichia coli from whole genome sequencing data. J Infect 88:191–193. doi:10.1016/j.jinf.2023.11.009 37992876

[16] Weis C, Cuénod A, Rieck B, Dubuis O, Graf S, Lang C, Oberle M, Brackmann M, Søgaard KK, Osthoff M, Borgwardt K, Egli A. 2022. Direct antimicrobial resistance prediction from clinical MALDI-TOF mass spectra using machine learning. Nat Med 28:164–174. doi:10.1038/s41591-021-01619-9 35013613

[17] Chang RYK, Nang SC, Chan H-K, Li J. 2022. Novel antimicrobial agents for combating antibiotic-resistant bacteria. Adv Drug Deliv Rev 187:114378. doi:10.1016/j.addr.2022.114378 35671882

[18] Diao Z, Han D, Zhang R, Li J. 2022. Metagenomics next-generation sequencing tests take the stage in the diagnosis of lower respiratory tract infections. J Adv Res 38:201–212. doi:10.1016/j.jare.2021.09.012 35572406 PMC9091713

[19] Chen H, Zhan M, Liu S, Balloux F, Wang H. 2024. Unraveling the potential of metagenomic next-generation sequencing in infectious disease diagnosis: challenges and prospects. Sci Bull Sci Found Philipp 69:1586–1589. doi:10.1016/j.scib.2024.04.033 38670851

[20] Giovagnorio F, De Vito A, Madeddu G, Parisi SG, Geremia N. 2023. Resistance in Pseudomonas aeruginosa: a narrative review of antibiogram interpretation and emerging treatments. Antibiotics (Basel) 12:1621. doi:10.3390/antibiotics12111621 37998823 PMC10669487

[21] El-Sayed Ahmed MAE-G, Zhong L-L, Shen C, Yang Y, Doi Y, Tian G-B. 2020. Colistin and its role in the Era of antibiotic resistance: an extended review (2000-2019). Emerg Microbes Infect 9:868–885. doi:10.1080/22221751.2020.1754133 32284036 PMC7241451

[22] Saito N, Tsuchiya J, Itoga M, Okamura Y, Tsuyama H, Kimura M, Inoue F, Kimura T, Ozaki H, Tono Y, Minakawa S, Tomita H. 2024. Multiple blood culture sampling, proper antimicrobial choice, and adequate dose in definitive therapy supported by the antimicrobial stewardship team could decrease 30-day sepsis mortality rates. Infect Drug Resist 17:207–219. doi:10.2147/IDR.S445917 38283110 PMC10812706

[23] Fourgeaud J, Regnault B, Ok V, Da Rocha N, Sitterlé É, Mekouar M, Faury H, Milliancourt-Seels C, Jagorel F, Chrétien D, Bigot T, Troadec É, Marques I, Serris A, Seilhean D, Neven B, Frange P, Ferroni A, Lecuit M, Nassif X, Lortholary O, Leruez-Ville M, Pérot P, Eloit M, Jamet A. 2024. Performance of clinical metagenomics in France: a prospective observational study. Lancet Microbe 5:e52–e61. doi:10.1016/S2666-5247(23)00244-6 38048804

