# Supplementary material for: Performance and hypothetical clinical impact of an mNGS-based machine learning model for antimicrobial susceptibility prediction of five ESKAPEE bacteria
Source: Microbiol Spectr. 2025 Apr 17;13(6):e02592-24. doi: 10.1128/spectrum.02592-24 (PMC12131724; doi:10.1128/spectrum.02592-24)
Supplement: Supplemental Material — Supplemental methods; legends for Tables S1 to S5. [file spectrum.02592-24-s0001.pdf]

Performance and Hypothetical Clinical Impact of an mNGS-Based Machine Learning Model for Antimicrobial Susceptibility Prediction of five ESKAPEE Bacteria

Running Title: Clinical Impact of mNGS-based AST of ESKAPEE

Yaoguang Li [a b 1], Sizhen Liu [c 1], Peng Han [c]; Jun Lei [a b], Huifen Wang [a b], Weiwei Zhu [a b], Zihui Dong [a], Yize Zhang [a], Zhi Jiang [c], Beiwen Zheng [d *], Guanhua Rao [c *], Zujiang Yu [a b *], Ang Li [a *]

[a] Gene Hospital of Henan Province, Precision Medicine Center, the First Affiliated Hospital of Zhengzhou University, Zhengzhou 450052, China

[b] Department of Infectious Disease, the First Affiliated Hospital of Zhengzhou University, Zhengzhou 450052, China

[c] Genskey Medical Technology Co., Ltd., Beijing 102200, China

[d] Collaborative Innovation Center for Diagnosis and Treatment of Infectious Diseases, State Key Laboratory for Diagnosis and Treatment of Infectious Diseases, The First Affiliated Hospital, Zhejiang University School of Medicine, Hangzhou 310003, China

[*] Corresponding: Dr. Ang Li (lia@zju.edu.cn; +86-18625505593); Prof. Zujiang Yu (johnyuem@zzu.edu.cn); Dr. Guanhua Rao (gh.rao@genskey.com); Dr. Beiwen Zheng (mpio@163.com)

[1] These authors contributed equally to this work.

CONTENTS

MATERIALS AND METHODS 3
1. STUDY DESIGN AND POPULATION 3
2. PATHOGEN AND ANTIMICROBIAL SUSCEPTIBILITY IDENTIFICATION 4
2.1 Bacterial Culture and Culture-based AST 4
2.2 Clinical mNGS and mNGS-based AST 4
3. CLINICAL DATA COLLECTION 7
4. PERFORMANCE AND HYPHOTHETICAL CLINICAL IMPACT EVALUATION 8
4.1 Primary Study: Performance Evaluation 8
4.2 Secondary Study: Hypothetical Clinical Impact Evaluation 9
5. Statistical Analysis 10
LIST OF SUPPLEMENTAL TABLES 12

MATERIALS AND METHODS

1. STUDY DESIGN AND POPULATION

We retrospectively reviewed the data from patients who underwent clinical mNGS (metagenomic next-generation sequencing) during the hospitalization. These patients were enrolled from a previous observational study of clinical mNGS applications at the First Affiliated Hospital of Zhengzhou University. In the previous study, patients were suspected of having an infectious disease (with typical symptoms of infection and/or abnormal imaging findings and/or abnormal inflammatory markers). And they underwent clinical mNGS to identify the pathogens.

Between July 2021 and March 2023, clinical specimens from 1,342 patients were subjected to paired bacterial culture and clinical mNGS. Among these, we identified 288 patients with clinical mNGS results positive for the five ESKAPEE bacteria, including *Acinetobacter baumannii* (AB), *Klebsiella pneumoniae* (KP), *Pseudomonas aeruginosa* (PA), *Escherichia coli* (EC), and *Staphylococcus aureus* (SA).

In this study, we incorporated the sequencing data of clinical mNGS into the self-constructed mNGS-based antimicrobial susceptibility prediction model (mNGS-based AST) for analysis. Patients with available mNGS-based AST results for the five ESKAPEE bacteria were initially included. Under the assumption that the results of mNGS-based AST would typically be obtained during the clinical management of the patients, we evaluated the utility of mNGS-based AST for enrolled patients from two perspectives: performance and hypothetical clinical impact. A schematic of the patient inclusion/exclusion workflow is shown in **Figure 1**.

For the primary study, we evaluated the performance of mNGS-based AST. Antimicrobial susceptibility results of 113 confirmed bacterial strains were reviewed, including 39 strains of AB, 36 strains of KP, 20 strains of PA, 7 strains of EC and 11 strains of SA. All of them had available results from both culture-based traditional antimicrobial susceptibility testing (culture-based AST) and mNGS-based AST.

For the secondary study, we excluded patients whose medical records over the follow-up period (7 days) were incomplete. We yielded two cohorts according to bacterial culture results to evaluate the hypothetical clinical impact of the mNGS-based AST model, including the culture-positive group (n = 78) and the culture-negative group (n = 36). A schematic of the patient inclusion/exclusion workflow is shown in **Figure 1**.

2. PATHOGEN AND ANTIMICROBIAL SUSCEPTIBILITY IDENTIFICATION

2.1 Bacterial Culture and Culture-based AST

Vitek 2 Compact system (bioMérieux, France) was used for bacterial identification and culture-based AST according to the manufacturer's instructions.

Fresh pure cultures were used for the assay. 0.85% NaCl or deionized water was used to prepare the appropriate concentration of the identified bacterial suspension. The concentration was corrected using the DensiCHEK plus turbidimeter (BioMérieux, France) to ensure that the bacterial suspension had a concentration of 0.5 McNeil's turbidity.

The antimicrobial susceptibility of isolates was tested by the VITEK 2 system using AST-N335, AST-GN09 and AST-GP67 test cards (Oxoid, England) according to the Clinical and Laboratory Standards Institute (CLSI) (M100-S30 and M45-A3) and European Committee on Antimicrobial Susceptibility Testing (EUCAST) (v10.0) breakpoint tables, except for tigecycline and cefoperazone/sulbactam, for which the U.S. Food and Drug Administration (FDA) breakpoint tables were used.

Antimicrobial susceptibility was reported as S (susceptible, susceptible at normal dosing), I (intermediate, susceptible at increased exposure) or R (resistant, resistant to the agent).Values were validated by concurrently testing the following ATCC quality control reference strains: *Escherichia coli* ATCC 25922, *Pseudomonas aeruginosa* ATCC 27853, *Staphylococcus aureus* ATCC 29213, and *Staphylococcus aureus* ATCC 25923.

2.2 Clinical mNGS and mNGS-based AST

2.2.1 Sample processing and sequencing

mNGS-based AST leveraged the same sample processing and sequencing stage as clinical mNGS.

The microbes were broken by fine grinding method. DNA from all specimens, including the negative batch, was extracted by Genskey Micro DNA Kit (1901, Genskey) and quantified by Qubit dsDNA HS Assay Kit. The DNA library was constructed using NGS library construction kit (Enzyatics). Agilent 2100 Bioanalyzer (Agilent Technologies, Santa Clara, USA) was used to assess the quality of DNA libraries.

Specimens with DNA concentration < 1 ng/uL were subjected to additional PCR. DNB

was prepared by thermal denaturation and mixing of the library. The DNA nanospheres were loaded onto the sequencing chip and sequenced using MGISEQ-200 sequencing platform (MGI, Shenzhen, China).

2.2.2 Bioinformatics analysis

2.2.2.1 Bioinformatics analysis for clinical mNGS

The raw reads underwent quality control processes using fastp (v 0.23.2), which included removal of low-quality, excessively short, redundant, and adapter sequences. The in-house script DCfilter was used to remove low-complexity sequences. As a result of these procedures, the raw reads were converted into clean reads.

Clean reads aligned to the human reference assembly (Human GRCh38/hg38) were excluded using bowtie2 (v.2.3.5.1).The remaining reads were considered to be of potential microbial origin.

To guarantee precise annotation, we integrated four databases: NCBI RefSeq database (v.20221231), FDA-ARGOS (v1.0), Genome Taxonomy Database (release206), and NCBI GenBank database (v.20221231), to form a comprehensive pathogen reference genome database for pathogens. We used BWA (0.7.17-r1198-dirty) to align the reads that may have originated from microbes to this merged database, producing raw alignment outcomes. Using the in-house script pathogen_summary.py, we classified the aligned sequences and quantified their consistency and coverage.

Receiver operator characteristic (ROC) curves were generated to evaluate test outcomes from both bioinformatically simulated and clinically derived samples across different systems. The in-house script lucy.py was then used to generate final reports, which included sample information, interpretation results, and the original detected results for pathogen detection, in order to align pathogen reports with clinical diagnoses.

2.2.2.2 Bioinformatics analysis for mNGS-based AST

To establish a machine learning model capable of predicting antimicrobial susceptibility information for focused pathogens within mNGS data, we collected comprehensive genomic sequences and corresponding antimicrobial susceptibility profiles for AB, KP, PA, EC and SA from NCBI and PATRIC databases. The process involved assembling a composite resistance gene database by integrating information from CRAD, ARDB, and Resfinder. Then, the assembled contigs of individual bacterial genomes were aligned with this custom-built database to determine the presence of resistant genes.

The subsequent step involved using a ten-fold cross-validation least absolute shrinkage and selection operator (LASSO) regression model to compute correlation coefficients between genotypic elements and phenotypic antimicrobial susceptibilities. This helped to identify and select candidate resistance features that are significantly relevant. The positive detection rates of the candidate traits for each unique pairing of pathogen and antimicrobials were calculated within the training dataset. Features that did not meet a predefined threshold were then systematically filtered out.

During the feature selection process, coefficient of variation curves was plotted to pinpoint the juncture at which the minimal variability occurred among the selected features, thereby enabling the assignment of weights reflective of their respective contributions. Distinct models were formulated for each bacterium in relation to each antibacterial, generating ROC curves and calculating the Area Under the Curve (AUC) values as evaluative metrics for the model's predictive accuracy.

Based on these results, a rigorous manual curation of the features ensued, involving the elimination of non-vital characteristics and retention of those having substantial impact on model performance. This process resulted in the derivation of a model with the highest AUC value, which we utilized in subsequent investigations and applications.

Due to the significantly shorter reads of mNGS sequencing data compared to the contigs derived from whole genome assemblies, direct application of models built on single bacterial genomes to mNGS data requires setting thresholds during the alignment process of mNGS-derived sequence reads to infer pathogen antimicrobial resistance or susceptibility profiles. A standard approach to predicting antimicrobial resistance is to compare each predicted value to a predefined critical value. The calculation of such scores follows this formula:

$$Score = \sum_{i=1}^{n} Genefamily\_Wi \ || \ AMR\_feature\_Wi$$

Here, *AMR_feature_Wi* represents the weight assigned to each antimicrobial resistance trait, while *Genefamily_Wi* represents the weights attributed to categorized gene families. The variable n represents the total number of AMR gene families under consideration.

When applying the antimicrobial susceptibility prediction model to clinical specimens, the presence of inherent sequencing depth heterogeneity underscores the necessity for a strategy that ensures precise identification of species associated with detected

antimicrobial resistance genes (ARGs). This strategy involves the utilization of species-specific k-mers in conjunction with ancillary methodologies, such as evaluating potential species origin and conducting copy number range analyses. Upon encountering a pathogen potentially harboring ARGs, the relative copy numbers of each ARG per pathogenic species are computed. These copy numbers are subsequently assessed to determine whether they fall within the established normal range of ARG gene copy number variations derived from a training dataset. ARGs residing within this normative spectrum are provisionally assigned to the corresponding species. However, in instances where an ARG exhibits copy number ranges compatible with multiple pathogens, definitive species attribution is achieved by leveraging the specificity of k-mers associated with the ARG in question.

In this study, we incorporated the sequencing data from prior clinical mNGS testing into the newly established predication model. Pathogens that met the required sequencing depth threshold were included. If any resistance feature included in optimal model was detected, antimicrobial susceptibility was reported as "R-predicted". If no such features were found, susceptibility determinations were based on the AUC value of the optimal model, using 0.9 as the threshold: for antibacterial-bacterium pairs with an AUC value exceeding 0.9, "S-predicted" was reported, conversely, "Not-predicted" was reported.

3. CLINICAL DATA COLLECTION

The following clinical data of the enrolled patients were retrospectively collected by reviewing the Electronic Medical Record System and the Electronic Medical Prescription System of the First Affiliated Hospital of Zhengzhou University:

(a) General hospitalization information.

(b) Routine test results, including white blood cell count (WBC), neutrophil count (NEUT), C-reactive protein (CRP) and procalcitonin (PCT).

(c) Etiological examination results and turnaround time, including results of bacterial culture, culture-based AST and clinical mNGS.

(d) Therapy: antibacterial use on the day of sampling (D0) and in the following 7 days (D1-D7).

4. PERFORMANCE AND HYPHOTHETICAL CLINICAL IMPACT EVALUATION

4.1 Primary Study: Performance Evaluation

Inclusion criteria:

(a) Five ESKAPEE bacteria were detected by both bacterial culture and clinical mNGS, including AB, KP, PA, EC and SA.

(b) Available results were obtained from both culture-based AST and mNGS-based AST for the five ESKAPEE bacteria.

Exclusion criteria:

(a) Five ESKAPEE bacteria were not detected by bacterial culture or clinical mNGS.

(b) No available results were obtained from culture-based AST or mNGS-based AST for the five ESKAPEE bacteria.

Methods and outcomes:

The results of culture-based AST, initially reported as a trichotomous outcome (S, I or R, as indicated above), were converted to dichotomous results (S or I/R) to facilitate comparisons with the dichotomous outcomes from mNGS-based AST (S-predicted or R-predicted). When both methods indicated susceptibility to a particular antimicrobial agent for a specific strain (S vs. S-predicted), this agreement was labelled as true susceptibility. Similarly, when both methods indicated a lack of susceptibility (I/R vs. R-predicted), this concordance was labelled as true resistance. In cases of discrepancy, major error (ME, S vs. R-predicted) and very major error (VME, I/R vs. S-predicted) were defined to indicate the cases. If mNGS-based AST failed to generate the antimicrobial susceptibility results to an antimicrobial, the item was classified as Unpredicted. Entries were further categorized as Unpredicted-S or Unpredicted-R/I based on the culture-based AST results.

Using culture-based AST results as the gold standard, we evaluated the performance of mNGS-based AST. For the items that results were available from mNGS-based AST, we calculated accuracy, positive predictive value (PPV), and negative predictive value (NPV). Accuracy was defined as the proportion of true resistance and true susceptibility items to predictable items. PPV was defined as the proportion of true resistance items to the sum of true resistance and ME items. NPV was defined as the proportion of true susceptibility items to the sum of true susceptibility and VME items. For all items, we

calculated the predictable ratio, defined as the proportion of predictable cases to total number of items.

Additionally, the turnaround time of clinical mNGS (between sampling and obtaining available reports) was collected from the Electronic Medical Record System of the First Affiliated Hospital of Zhengzhou University. The turnaround time for mNGS-based AST was considered equivalent to that of clinical mNGS. The differences in turnaround time between mNGS-based and culture-based AST were assessed.

4.2 Secondary Study: Hypothetical Clinical Impact Evaluation

4.2.1 Hypothetical Clinical Impact Evaluation in culture-positive group

Inclusion criteria:

(a) Five ESKAPEE bacteria were detected by both bacterial culture and clinical mNGS, including AB, KP, PA, EC and SA.

(b) Available results were obtained from both culture-based AST and mNGS-based AST for the five ESKAPEE bacteria.

(c) Medical records were complete and available in a follow-up period (7 days).

Exclusion criteria:

(a) Five ESKAPEE bacteria were not detected by bacterial culture or clinical mNGS.

(b) No available results were obtained from culture-based AST or mNGS-based AST for the five ESKAPEE bacteria.

(c) Medical records were not complete or available in a follow-up period (7 days).

Methods and outcomes:

During real-world clinical management, specimens were collected and sent for bacterial culture and clinical mNGS on D0. Upon receiving clinical mNGS results on D1, clinicians could make initial adjustments referring to the mNGS findings. Further adjustments were made from D2 to D7, incorporating bacterial culture results and patient response over this period **(Figure 2B(a))**.

We aimed to assess the clinical benefits of the model in guiding therapy adjustments. To achieve this, we conducted a simulated management experiment, assuming that mNGS-based AST results were available on D1 along with clinical mNGS results. We analysed the model's hypothetical clinical impact based on recommendations for the antibacterial

therapy administered to patients on D1. Two experienced physicians provided therapeutic recommendations independently based on mNGS-based AST or culture-based AST. These recommendations were categorized into three types: de-escalation (switching to narrower-spectrum antibacterials), escalation (switching to broader-spectrum antibacterials) and maintenance (continuing the current therapy). The cases with inconsistent judgments were re-evaluated through a case review process.

In simulated management, we primarily assessed whether the confirmed pathogenic bacterial strains were resistant to antibacterials administered on D1 according to mNGS-based AST or culture-based AST results, respectively. For patients in the culture-positive group, we compared the physicians' recommendations based on each method. Cases where the recommendations were consistent were considered that mNGS-based AST had a positive clinical impact, as mNGS-based AST enabled earlier therapy adjustments (D1 vs. D2-7) **(Figure 2B(b))**.

4.2.2 Hypothetical Clinical Impact Evaluation in culture-negative group

Inclusion criteria:

(a) Five ESKAPEE bacteria were detected by clinical mNGS, including AB, KP, PA, EC and SA. Five ESKAPEE bacteria were not detected by bacterial culture.

(b) Available results were obtained from mNGS-based AST for the five ESKAPEE bacteria.

(c) Medical records were complete and available in a follow-up period (7 days).

Exclusion criteria:

(a) Five ESKAPEE bacteria were not detected by clinical mNGS.

(b) No available results were obtained from mNGS-based AST for the five ESKAPEE bacteria.

(c) Medical records were not complete or available in a follow-up period (7 days).

Methods and outcomes:

For patients in culture-negative group, we assessed the proportion of those who could receive available recommendations despite the absence of culture-based results **(Figure 2B(c))**.

5. Statistical Analysis

Continuous variables are expressed as median (interquartile range, IQR) or mean (standard deviation, SD). For normally distributed variables, independent *t-test* was performed with a significance level set of $\alpha = 0.05$. Categorical variables are presented as frequencies and percentages. Statistical analysis was conducted using SPSS (version 28.0, IBM Corp. USA).

LIST OF SUPPLEMENTAL TABLES

sTable 1. Performance of mNGS-based AST

(As shown in Sheet 1 of *supplemental tables.xlsx*)

sTable 2. Detected AMR Features

(As shown in Sheet 2 of *supplemental tables.xlsx*)

sTable 3. Performance of mNGS-based AST (at the bacterium-specific level)

(As shown in Sheet 3 of *supplemental tables.xlsx*)

sTable 4. Performance of mNGS-based AST (at the antibacterial-specific level)

(As shown in Sheet 4 of *supplemental tables.xlsx*)

sTable 5. Time Efficiency of mNGS-based AST

(As shown in Sheet 5 of *supplemental tables.xlsx*)
